# Dynamic physiological responses in obese and non-obese adults submitted to cardiopulmonary exercise test

**DOI:** 10.1371/journal.pone.0255724

**Published:** 2021-08-09

**Authors:** Bárbara de Barros Gonze, Thatiane Lopes Valentim Di Paschoale Ostolin, Alan Carlos Brisola Barbosa, Agatha Caveda Matheus, Evandro Fornias Sperandio, Antônio Ricardo de Toledo Gagliardi, Rodolfo Leite Arantes, Marcello Romiti, Victor Zuniga Dourado

**Affiliations:** 1 Department of Human Movement Sciences, Federal University of São Paulo (UNIFESP), Santos, São Paulo, Brazil; 2 Angiocorpore Institute of Cardiovascular Medicine, Santos, São Paulo, Brazil; 3 Lown Scholars Program–Harvard T.H. Chan School of Public Health, Boston, Massachusetts, United States of America; Scuola Superiore Sant’Anna, ITALY

## Abstract

**Purpose:**

Obese individuals have reduced performance in cardiopulmonary exercise testing (CPET), mainly considering peak values of variables such as oxygen uptake ($\dot{V}O_{2}$), carbon dioxide production ($\dot{V}CO_{2}$), tidal volume (Vt), minute ventilation ($\dot{V}E$) and heart rate (HR). The CPET interpretation and prognostic value can be improved through submaximal ratios analysis of key variables like $\mathrm{\Delta HR}/\Delta \dot{V}O_{2}$, $\Delta \dot{V}E/\Delta \dot{V}CO_{2}$, $\Delta \dot{V}C/\Delta \mathrm{linearized}$ $(\ln)\dot{V}E$ and oxygen uptake efficiency slope (OUES). The obesity influence on these responses has not yet been investigated. Our purpose was to evaluate the influence of adulthood obesity on maximal and submaximal physiological responses during CPET, emphasizing the analysis of submaximal dynamic variables.

**Methods:**

We analyzed 1,594 CPETs of adults (755 obese participants, Body Mass Index ≥ 30 kg/m^2^) and compared the obtained variables among non-obese (normal weight and overweight) and obese groups (obesity classes I, II and III) through multivariate covariance analyses.

**Result:**

Obesity influenced the majority of evaluated maximal and submaximal responses with worsened CPET performance. Cardiovascular, metabolic and gas exchange variables were the most influenced by obesity. Other maximal and submaximal responses were altered only in morbidly obese. Only a few cardiovascular and ventilatory variables presented inconsistent results. Additionally, Vt_max_, $\mathrm{Vt}/\dot{V}E$, Vt/Inspiratory Capacity, Vt/Forced Vital Capacity, Lowest $\dot{V}E/\dot{V}CO_{2}$, $\Delta \dot{V}E/\Delta \dot{V}CO_{2}$, and the y-intercepts of $\dot{V}E/\dot{V}CO_{2}$ did not significantly differ regardless of obesity.

**Conclusion:**

Obesity expressively influences the majority of CPET variables. However, the prognostic values of the main ventilatory efficiency responses remain unchanged. These dynamic responses are not dependent on maximum effort and may be useful in detecting incipient ventilatory disorder. Our results present great practical applicability in identifying exercise limitation, regardless of overweight and obesity.

## Introduction

Cardiopulmonary exercise test (CPET) presents indisputable clinical applicability for the diagnosis of exercise intolerance and its causes, as well to quantify cardiorespiratory fitness (CRF) [1, 2]. Therefore, the maximal pulmonary oxygen uptake ($\dot{V}O_{2\max}$) obtained at the end of a CPET with maximum effort is widely used. Obese individuals present decreased CRF, mainly caused by low pulmonary complacency and functional residual capacity [3] combined with a less pronounced chronotropic response during sustained effort [4].

The total duration of CPET, as well as $\dot{V}O_{2\max}$ and maximum heart rate (HR_max_), are inversely proportional to body mass index (BMI) [5]. Considering possible difficulties in reaching the maximum effort leading to interpretative limitation of the maximal incremental exercise variables (e.g., $\dot{V}O_{2\max}$, HR_max_, metabolic equivalent of task (MET), gas exchange rate (R) and the other variables registered at peak of effort), the CPET interpretation can be significantly improved by an evaluation of the tendency of submaximal relationships (e.g., anaerobic threshold (AT) values and lowest $\dot{V}E/\dot{V}CO_{2}$, while OUES and the aforementioned ratios and intercepts) obtained before the peak of the exercise.

Previously identified as determinants for prognosis and survival in several clinical situations [6], these submaximal relationships have been recommended in the evaluation of chronic diseases patients, mainly cardiorespiratory [7]. Also known as dynamic physiological responses, the main advantage of these variables is its linear behavior during the increment or workload, regardless achieving maximum effort [8, 9]. In addition, the evaluation of these submaximal variables is especially interesting in obese population, which present difficulties to achieve maximum effort [10, 11].

The higher slope of the $\Delta \mathrm{HR}/\Delta \dot{V}O_{2}$ relationship, for example, is an important indirect indicator of cardiovascular inefficiency and peripheral muscle disability. Similarly, ventilatory inefficiency can be measured either by the greater slope of the ratio between minute ventilation ($\dot{V}E$) per unit of carbon dioxide ($\dot{V}CO_{2}$) released ($\Delta \dot{V}E/\Delta \dot{V}CO_{2}$) or by the lowest point (known as nadir) of the quotient of $\Delta \dot{V}E/\Delta \dot{V}CO_{2}$ (lowest $\Delta \dot{V}E/\Delta \dot{V}CO_{2}$), which indicates increased respiratory dead space and/or hyperventilation. In addition, the slope of the relationship between tidal volume (Vt) and $\dot{V}E$ linearized by logarithmic transformation ($\Delta \mathrm{Vt}/\mathrm{\Delta ln}\dot{V}E$) translates the tachycardic pattern during exercise [1, 8]. The relationship between $\dot{V}O_{2}$ and $\dot{V}E$ logarithm in base 10 ($\log\dot{V}E$) ($\Delta \dot{V}O_{2}/\mathrm{\Delta log}\dot{V}E$) translates the oxygen uptake efficiency slope (OUES) [1, 8].

Although several variables obtained in maximal effort identify worse values in obese, there is considerable controversy over dynamic physiological responses. Limited evidence shows that both OUES and $\dot{V}E/\dot{V}CO_{2}$ present comparable values between obese and non-obese individuals [12, 13]. However, such evidence was obtained in studies with insufficient samples and inadequate statistical power and, hence, the association of obesity with these variables remains unknown. Therefore, we hypothesized that the main dynamic physiological responses of clinical interest do not suffer significant influence of obesity, maintaining their diagnostic and prognostic values unchanged. We aimed to evaluate the influence of adulthood obesity on maximal and submaximal physiological responses during CPET, emphasizing the analysis of submaximal dynamic variables.

## Materials and methods

### Participants and study design

We conducted a cross-sectional study with a convenience sample that retrospectively analyzed CPETs carried out between 2013 and 2018 from the database of the endocrinology outpatient clinic of a Cardiovascular Medicine Institute and from an ongoing Epidemiological Study. The EPIMOV Study is a prospective cohort study whose primary purpose is to investigate the association between low physical activity level and low physical fitness and the development of chronic diseases and conditions, especially cardiovascular and musculoskeletal diseases [14]. Participants were informed about the possible risks and discomforts of the evaluations and signed an Informed Consent Term. The Federal University of São Paulo Research Ethics Committee approved both the Epidemiological Study (#186.796/2013) and this study (#3.006.451/2017).

Inclusion criteria of the Epidemiological Study is the absence of previously diagnosed heart, lung or locomotor diseases and being capable of performing physical effort. We excluded individuals who presented difficulty in comprehend or perform the test, potentially lethal arrhythmias requiring early interruption of CPET, chest pain, use of β-blocker medication, submaximal effort, and operational problems during the CPET due to several factors, for example, mechanical failure (hardware or software).

### Clinical evaluation

History of health problems, physical activity level, risk factors for cardiovascular disease and prior use of medications were investigated through self-report. We consider as risk factors for cardiovascular diseases: age (men ≥ 45 years, women ≥ 55 years), family history of cardiovascular disease, systemic arterial hypertension, diabetes mellitus, dyslipidemia, current smoking, and physical inactivity (e.g., < 150 minutes of moderate-to-vigorous physical activity per week) [15].

### Anthropometric measures

Body mass (kg) and height (m) were measured in a scale with a stadiometer [16]. Then, we calculated BMI. Lastly, obesity was classified according to BMI (normal weight: 18.5–24.9 kg/m^2^; overweight: 25.0–29.9 kg/m^2^, obese class I: 30.0–34.9 kg/m^2^; obese class II: 35.0–39.9 kg/m^2^; obese class III: ≥ 40.0 kg/m^2^) [17].

### Cardiopulmonary exercise testing

The CPET was performed in a motorized treadmill (ATL, Inbramed, Porto Alegre, Brazil) following an individualized ramp protocol. Based on the age, sex, body mass, height, and physical activity level, the Inbramed software estimate $\dot{V}O_{2}$ maximum. Additionally, the same variables of each participant were considered to increase automatically speed and grade in a linear and individualized ramp during the test.

The test starts with speed and grade at 3 km/h and 0%, respectively. Before the effort until exhaustion, the subject remains 3 minutes at rest, allowing an initial evaluation of baseline measurements. Then, the maximal CPET aims to take the subject to exhaustion within the period of 8 and 12 minutes of exercise, followed by 3 minutes of recovery. When the subject reaches exhaustion, the CPET is interrupted by the subject’s request. However, the CPET may also be interrupted by the physician, e.g., signals of suggestive myocardial ischemia (ST-segment depression), chest pain, sudden drop in systolic blood pressure ≥ 20 mmHg, systolic blood pressure ≥ 250 mmHg, signs of respiratory failure, loss of coordination and mental confusion.

The entire test was also performed with a 12-lead electrocardiogram (C12X, COSMED, Italy) to monitor HR and possible abnormalities in the electrocardiographic tracing. Systolic and diastolic blood pressure (SBP; DBP) were obtained every two minutes during CPET and we calculated the double product (DP) (SBP x HR). A cardiologist responsible for the study evaluated the CPETs, as well as the electrocardiographs.

Throughout the CPET, perceived exertion regarding dyspnea and lower limb fatigue were assessed using the modified Borg scale [18]. Metabolic, cardiovascular, and ventilatory breath-to-breath responses were obtained with a gas analyzer (Quark PFT, COSMED, Pavona Albano, Italy) and filtered at 15-second intervals. When the subject achieved HR at the peak of exercise ≥ 85% of the predicted for age (220 –age) [19] or gas exchange rate (R) ≥ 1.0 or a $\dot{V}O_{2}$ plateau, we considered the equivalent value of $\dot{V}O_{2}$ as the peak effort [1]. The $\dot{V}O_{2\max}$ was defined as the average value obtained during the last 15 seconds of CPET and expressed as absolute value (mL^.^ min^-1^), relative to the body mass (mL^.^ min^-1.^ kg) and percentage of the predicted value (%pred) [8]. We considered $\dot{V}O_{2\max}$ < 83% $\dot{V}O_{2\mathrm{pred}}$ as exercise intolerance.

**Fig 1:**
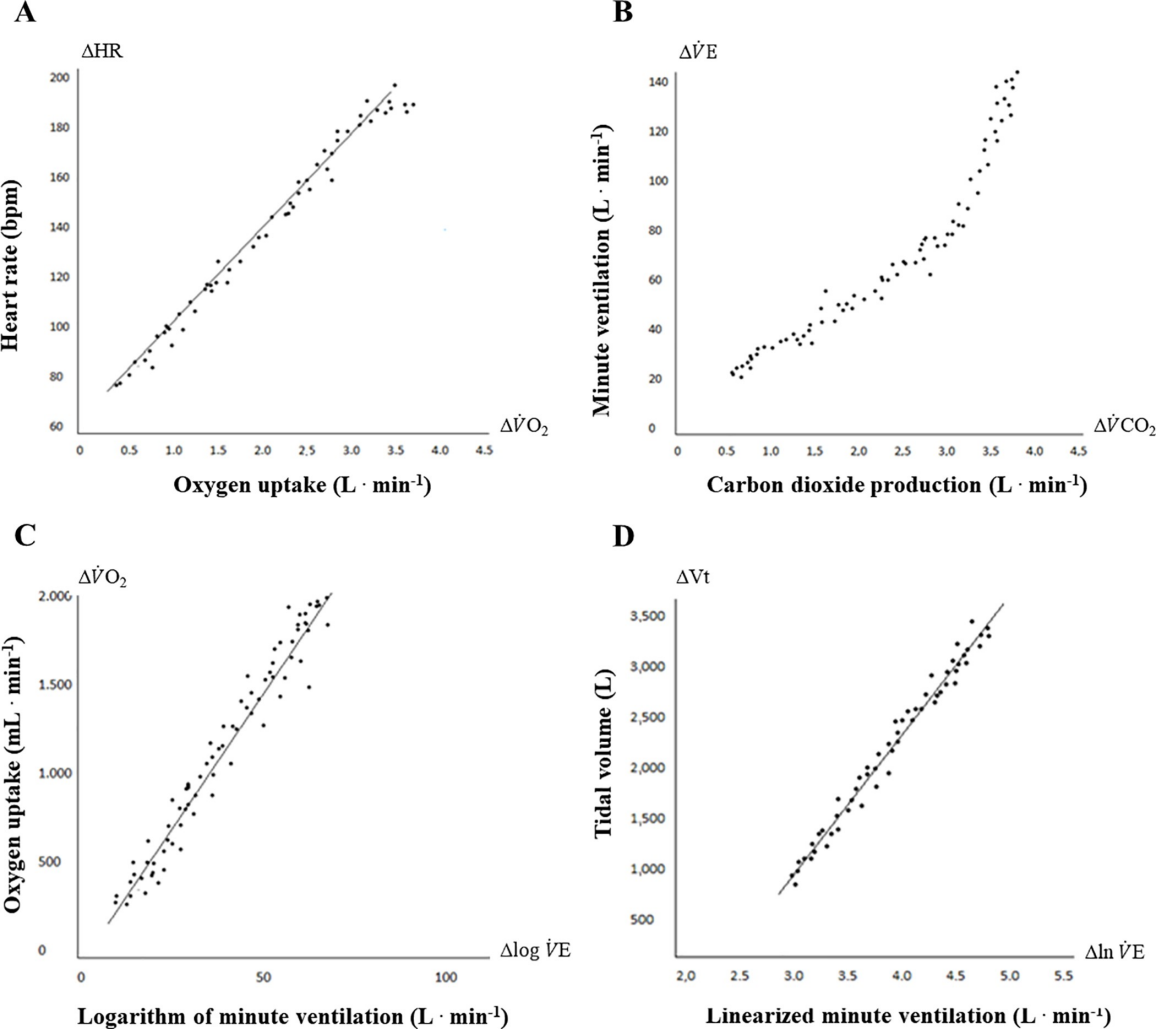
Procedure used to establish the dynamic physiological responses. (A) Cardiovascular efficiency ($\mathrm{\Delta HR}/\Delta \dot{V}O_{2}$). (B) Ventilatory efficiency ($\Delta \dot{V}E/\Delta \dot{V}CO_{2}$). (C) Oxygen uptake efficiency slope ($\Delta \dot{V}O_{2}/\mathrm{\Delta log}\dot{V}E$). (D) Ventilatory pattern ($\mathrm{\Delta Vt}/\mathrm{\Delta ln}\dot{V}E$).

The oxygen pulse (PuO_2_) was assessed through the ratio between $\dot{V}O_{2}$ and HR ($\Delta \dot{V}O_{2}/\Delta \mathrm{HR}$). We also established the ratios between key variables, e.g., $\dot{V}E/\dot{V}O_{2}$ and $\dot{V}E/\dot{V}CO_{2}$. The $\dot{V}O_{2}$ equivalent to anaerobic threshold (AT) was obtained by using the ventilatory method (e.g., the first nonlinear increment of $\dot{V}E/\dot{V}O_{2}$ with simultaneous $\dot{V}E/\dot{V}CO_{2}$ stabilization) combined with the gas exchange method based on visual inspection at the point of inflection of $\dot{V}CO_{2}/\dot{V}O_{2}$ (V slope) [1]. The dynamic submaximal responses analyzed were obtained by linear regressions as previously described [8]: $\mathrm{\Delta HR}/\Delta \dot{V}O_{2}$, $\Delta \dot{V}E/\Delta \dot{V}CO_{2}$ and $\Delta \mathrm{Vt}/\Delta \ln\dot{V}E$ and OUES. We provided an example for these relationships’ kinetic behavior as mentioned earlier obtained in an asymptomatic adult population, as can be seen in Fig 1.

Thus, $\dot{V}O_{2}$ max, HR max, MET and R were considered maximal responses. Additionally, ventilatory, cardiovascular and gas exchange responses were registered at the peak of effort and, hence, also considered as maximal responses. In turn, the submaximal responses from CPET were AT values and Lowest $\dot{V}E/\dot{V}CO_{2}$, while OUES and the ratios as mentioned above and intercepts were the main dynamic physiological responses evaluated. Lastly, exercise duration, maximum treadmill speed and grade were registered for further analysis.

### Statistical analysis

We used the SPSS program, version 24 (SPSS IBM Corp., Armonk, NY, USA), for all statistical analysis performed. The data were analyzed descriptively. Then, we applied the Kolmogorov-Smirnov test to evaluate data normality. In addition, we realized the analysis of histograms, Q-Q graphs, curve symmetry and standard error. According to the normal or non-normal distribution of the variables, the data were described as mean ± standard deviation or as median (interquartile range) for continuous variables, while categorical variables were expressed as frequencies and percentages.

Our sample was divided into obese (normal weight and overweight) and non-obese (obesity classes I, II and III) groups. We compared obese and non-obese groups through the *Student*’s t-test and χ^2^ test for continuous and categorical variables, respectively.

We performed a series of multivariate analysis of variance to evaluate the effect of obesity on CPET variables. The CPET variables were considered outcomes, while BMI as the main predictor and other variables as covariates. Therefore, multivariate models were adjusted by age, sex, and cardiovascular risk factors (e.g., arterial hypertension, diabetes, dyslipidemia, and physical inactivity).

The sample was calculated based on the size of the standardized effect between obese and non-obese groups. The probability of error α was set at 5% with a statistical power of 80%. We considered a low standardized effect size (< 0.25) as our aim was to evaluate a sufficient sample to do not observe differences between non-obese (normal weight and overweight) and obese (classes I, II and III). Therefore, the minimum number of individuals was, at least, 253 per group.

## Results

**Fig 2:**
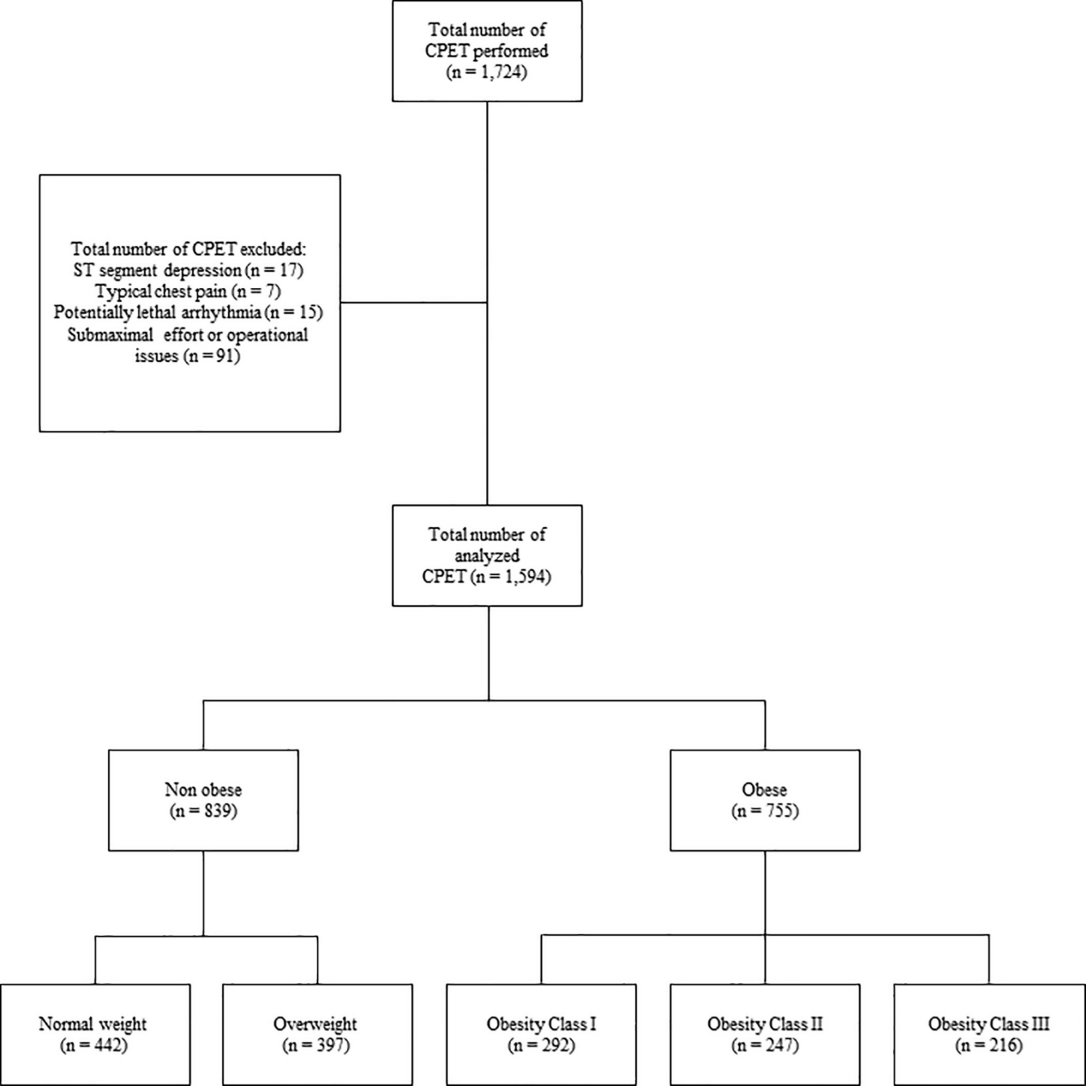
Flowchart.

From 1,724 CPETs, 1,594 were eligible for analysis (839 and 755 were non-obese and obese participants, respectively), as can be seen in Fig 2.

We considered 442 normal weight and 397 overweight participants as non-obese group. The obese group was composed by 292, 247 and 216, respectively, classes I, II and III. As expected, we found significant differences for anthropometric and cardiovascular risk variables when compared obese and non-obese participants. On average, obese group was mainly classified as obesity class II. Except of current smoking, obese participants had a higher proportion of cardiovascular disease risk factors in comparison with non-obese group (Table 1).

**Table 1:** General characteristics of the sample stratified as non-obese and obese (n = 1,594).

	Non-obese	Obese
	(n = 839)	(n = 755)
Age (years)	39 ± 14	42 ± 13*
**Sex, n (%)**		
Female	426 (50.8)	463 (61.3)
Male	413 (49.2)	292 (38.7)*
Body Mass (kg)	69.0 ± 11.8	103.7 ± 19.9*
Height (m)	1.66 ± 0.10	1.65 ± 0.10
Body Mass Index (kg/m^2^)	24.7 ± 2.8	37.5 ± 5.6*
Exercise intolerance (%)	13.3	27.1*
**Risk Factors for Cardiovascular Disease (%)**		
Arterial hypertension^¥^	8.4	25.6*
Diabetes Mellitus^¥^	4.9	13.0*
Dyslipidemia^¥^	16.3	27.1*
Physical inactivity^¥^	33.3	68.2*
Current smoking^¥^	9.9	11.9

Data were expressed as mean ± standard deviation and frequency (percentage) for continuous and categorical variables, respectively.

¥ Data obtained by self-report.

*p ≤ 0.05: Non-obese vs. Obese.

Obese participants presented the worst performance in most of the variables evaluated, when the indices were not adjusted by main confounders (t-test). Only few measures (lowest value of $\dot{V}E/\dot{V}CO_{2}$ and $\Delta \dot{V}E/\Delta \dot{V}CO_{2}$) were not significantly different between groups (Table 2).

**Table 2:** Physiological responses to cardiopulmonary exercise testing according to non-obese and obese groups (n = 1,594).

	Non-obese	Obese
	(n = 839)	(n = 755)
Exercise duration (min)	9.9 ± 1.5	8.5 ± 3.1 *
**Maximal responses**		
***Metabolic***		
$\dot{V}O_{2}$ (mL^.^ min^-1^)	2562.3 ± 935.1	2348.5 ± 758.7*
$\dot{V}O_{2}$ (mL^.^ min^-1.^ kg)	36.9 ± 11.0	22.8 ± 6.7*
$\dot{V}O_{2}$ (% pred)	103.9 ± 20.7	92.7 ± 17*
MET	10.4 ± 3.1	6.5 ± 1.9*
R ($\dot{V}CO_{2}/\dot{V}O_{2}$)	1.16 ± 0.12	1.10 ± 0.12*
***Ventilatory***		
$\dot{V}E$ max. (L^.^ min^-1^)	84.9 ± 31.9	72.6 ± 24.9*
$\dot{V}E/\mathrm{MVV}$ (L^.^ min^-1^)	51.3 ± 19.8	57.4 ± 17.2*
$\dot{V}E/\mathrm{MVV}$ (%)	39.0 ± 16.3	44.9 ± 13.2*
Vt max (L)	2.0 ± 0.6	1.9 ± 0.6*
Rf (ipm)	40.5 ± 8.2	37.8 ± 7.1*
Vt/IC	0.63 ± 0.12	0.60 ± 0.14*
Vt/FVC	60.9 ± 16.3	55.0 ± 13.2*
***Cardiovascular***		
HR (bpm)	170.8 ± 16.3	159.3 ± 17.7*
HR (% pred)	94.4 ± 6.5	89.4 ± 8.5*
HR reserve (bpm)	11 ± 16	19 ± 16*
HR recovery (bpm^.^ 1min^-1^)	30.8 ± 18.4	25.9 ± 17.8*
PuO_2_ (mL^.^ bpm^.-1^)	40.7 ± 12.9	46.0 ± 16.6*
SBP (mmHg)	175 ± 25	181 ± 26*
DBP (mmHg)	83 ± 9	88 ± 24*
DP (mmHg^.^ bpm)	29.985 ± 5.184	28.899 ± 5.400*
***Gas Exchange***		
PETCO_2_ (mmHg)	40.3 ± 4.8	41.2 ± 4.3*
PETO_2_ (mmHg)	112.1 ± 6.1	109.7 ± 5.0*
$\dot{V}E/\dot{V}CO_{2}$	29.4 ± 4.6	28.5 ± 3.6*
$\dot{V}E/\dot{V}O_{2}$	34.0 ± 12.4	30.9 ± 4.5*
**Submaximal responses**		
AT (mL^.^ min^-1^)	1,726.7 ± 710.9	1,609.5 ± 508.9*
AT (% $\dot{V}O_{2}$ peak)	67.3 ± 12.1	69.3 ± 9.7*
AT (% $\dot{V}O_{2}$ peak pred)	70.1 ± 20.3	64.0 ± 13.6*
Lowest $\dot{V}E/\dot{V}CO_{2}$	26.5 ± 3.3	26.6 ± 3.0
**Dynamic physiological responses**		
$\Delta \dot{V}E/\Delta \dot{V}CO_{2}$ (L^.^ min^-1.^ L^.^ min^-1^)	25.6 ± 4.0	25.4 ± 3.7
$\mathrm{\Delta HR}/\Delta \dot{V}O_{2}$ (bpm^.^ L^.^ min.^-1^)	46.0 ± 16.6	40.7 ± 12.9*
$\Delta \mathrm{Vt}/\mathrm{\Delta ln}\dot{V}E$	0.7 ± 0.2	0.7 ± 0.2*

Data were expressed as mean ± standard deviation.

*p ≤ 0.05: Non-obese vs. Obese.

$\dot{V}O_{2}$: Pulmonary oxygen uptake; $\dot{V}CO_{2}$: Carbon dioxide production; MET: Metabolic Equivalent of Task; AT: Anaerobic threshold; R: Gas exchange rate; $\dot{V}E$: Minute ventilation; MVV: Maximum voluntary ventilation; VR: Ventilatory reserve; Vt: Tidal volume; Rf: Respiratory frequency; ipm: Inspiration per minute; IC: Inspiratory capacity; FVC: Forced vital capacity; HR: Heart rate; bpm: Beat per minute; PuO_2_: Oxygen pulse; SBP: Systolic blood pressure; DBP: Diastolic blood pressure; PETO_2_: End expiratory oxygen pressure; PETCO_2_: Carbon dioxide end expiratory pressure; $\ln\dot{V}E$: Linearized ventilation; OUES: Oxygen uptake efficiency slope.

Similarly, after we performed a multivariate analysis, obesity remained negatively influencing most of the 40 physiological variables investigated. When higher BMI, lesser CPET duration and increment (treadmill speed and grade). Cardiovascular variables, for instance, HR_max_, HR_% pred_, HR_reserve_ and $\Delta \mathrm{HR}/\Delta \dot{V}O_{2}$, and the metabolic variables $\dot{V}O_{2\max}$, $\dot{V}O_{2\%\;\mathrm{pred}}$, MET and R presented the similar behavior (**Table 3**).

**Table 3:** According to normal weight, overweight and obesity groups (n = 1,594), physiological responses to cardiopulmonary exercise testing.

	Normal weight	Overweight	Obesity Class I	Obesity Class II	Obesity Class III
	(n = 442)	(n = 397)	(n = 292)	(n = 247)	(n = 216)
Exercise duration (min)	10.25 ± 1.52 ^b^^c^^d^^e^	9.65 ± 1.57 ^a^^d^^e^	9.16 ± 1.69 ^a^^e^	8.81 ± 4.85 ^a^^b^^e^	7.47 ± 1.77 ^a^^b^^c^^d^
Maximum speed (km/h)	9.2 ± 4.7 ^c^^d^^e^	8.5 ± 5.8 ^d^^e^	7.1 ± 4.5 ^a^	6.2 ± 0.9 ^a^^b^	5.6 ± 0.9 ^a^^b^^c^
Maximum grade (%)	9.7 ± 3.1 ^b^^c^^d^^e^	8.6 ± 1.9 ^a^^d^^e^	7.8 ± 1.7 ^a^^d^^e^	6.9 ± 1.7 ^a^^b^^c^^e^	5.6 ± 1.5 ^a^^b^^c^^d^
**Maximal responses**					
***Metabolic***					
$\dot{V}O_{2}$ (mL^.^ min^-1^)	2.530 ± 921 ^b^^c^^d^^e^	2.592 ± 948 ^a^	2.393 ± 893 ^a^	2.257 ± 661 ^a^	2.385 ± 665 ^a^
$\dot{V}O_{2}$ (mL^.^ min^-1.^ kg)	39.6 ± 11.1 ^b^^c^^d^^e^	33.8 ± 10.2 ^a^^c^^d^^e^	26. 5 ± 7.9 ^a^^b^^d^^e^	21.6 ± 4.8 ^a^^b^^c^^e^	19.2 ± 3.8 ^a^^b^^c^^d^
$\dot{V}O_{2}$ (% pred)	105 ± 21 ^c^^d^^e^	102 ± 19 ^d^^e^	97 ± 18 ^a^	90 ± 16 ^a^^b^	89 ± 13 ^a^^b^
MET	11.2 ± 3.1 ^b^^c^^d^^e^	9.6 ± 2.9 ^a^^c^^d^^e^	7.5 ± 2.2 ^a^^b^^d^^e^	6.1 ± 1.3 ^a^^b^^c^^e^	5.5 ± 1.1 ^a^^b^^c^^d^
R ($\dot{V}CO_{2}/\dot{V}O_{2}$)	1.1 ± 0.1 ^c^^d^^e^	1.1 ± 0.1 ^d^^e^	1.1 ± 0.1 ^a^^d^^e^	1.1 ± 0.1 ^a^^b^^c^^e^	1.0 ± 0.1 ^a^^b^^c^^d^
***Ventilatory***					
$\dot{V}E$ máx. (L^.^ min^-1^)	84.6 ± 31.2	85.0 ± 32.5 ^d^^e^	75.8 ± 29.3	70.0 ± 21.5 ^b^	71.1 ± 29.3 ^b^
$\dot{V}E/\mathrm{MVV}$ (L^.^ min^-1^)	52.0 ± 19.5	50.6 ± 20.1 ^d^^e^	53.5 ± 18.7	59.3 ± 16 ^b^	60.3 ± 15.5 ^b^
$\dot{V}E/\mathrm{MVV}$ (%)	39.2 ± 15.7	38.7 ± 17.0^d^	42.5 ± 15.7	46.3 ± 11.0 ^b^	46.4 ± 11.4
Vt máx. (L)	2.05 ± 0.63	2.12 ± 0.64	1.98 ± 0.65	1.93 ± 0.59	1.90 ± 0.58
Rf (ipm)	41 ± 8 ^d^^e^	39 ± 8 ^d^	38 ± 6	37 ± 7 ^a^^b^	38 ± 7 ^a^
Vt/IC	0.62 ± 0.12	0.65 ± 0.12	0.62 ± 0.13	0.60 ± 0.14	0.58 ± 0.14
Vt/FVC	0.49 ± 0.08	0.51 ± 0.08	0.53 ± 0.08	0.55 ± 0.10	0.55 ± 0.10
***Cardiovascular***					
HR (bpm)	173 ± 14 ^c^^d^^e^	168 ± 17^c^^d^^e^	161 ± 17 ^a^^b^^d^^e^	158 ± 17 ^a^^b^^c^	157 ± 17 ^a^^b^^c^
HR (% pred)	94 ± 5 ^c^^d^^e^	94 ± 7 ^c^^d^^e^	92 ± 8 ^a^^b^^d^^e^	88 ± 8 ^a^^b^^c^	86 ± 8 ^a^^b^^c^
HR reserve (bpm)	10 ± 15 ^d^^e^	10 ± 17 ^d^^e^	13 ± 13 ^d^^e^	21 ± 17 ^a^^b^^c^	24 ± 16 ^a^^b^^c^
HR recovery (bpm^.^ 1 min^-1^)	31 ± 18 ^e^	29 ± 17	25 ± 14	27 ± 17	25 ± 22 ^a^
PuO_2_ (mL^.^ bpm.^-1^)	14.4 ± 4.9 ^b^^c^^d^^e^	15.4 ± 5.8 ^a^^e^	14.7 ± 5.0 ^a^^e^	14.2 ± 3.7 ^a^^e^	15.1 ± 3.9 ^a^^b^^c^^d^
SBP max (mmHg)	170 ± 24 ^b^^c^^d^^e^	181 ± 26^a^	184 ± 25 ^a^	181 ± 25 ^a^	177 ± 27 ^a^
DBP max (mmHg)	81 ± 9 ^d^^e^	85 ± 9 ^c^	90 ± 3 ^a^^b^	87 ± 9	89 ± 9^a^
DP (mmHg^.^ bpm)	29,547 ± 5,047 ^b^^c^	30,438 ± 5,266 ^a^^e^	29,745 ± 5,284 ^a^^e^	28,771 ± 5,398	27,892 ± 5,361 ^b^^c^
***Gas Exchange***					
PETO_2_ (mmHg)	112.9 ± 4.8 ^b^^c^^d^^e^	111.3 ± 7.3 ^a^^d^^e^	110.3 ± 5.0 ^a^^e^	109.9 ± 4.6 ^a^^b^^e^	108.5 ± 5.1 ^a^^c^^d^
PETCO_2_ (mmHg)	39.9 ± 4.4 ^c^^d^^e^	40.7 ± 5.1 ^e^	41.1 ± 4.2 ^a^	41.0 ± 4.2 ^a^	41.5 ± 4.7 ^a^^b^
$\dot{V}E/\dot{V}CO_{2}$	29.9 ± 5.1 ^c^^d^^e^	29.9 ± 3.9 ^a^^e^	28.7 ± 3.8 ^a^	28.6 ± 3.4 ^a^	28.2 ± 3.6 ^a^^b^
$\dot{V}E/\dot{V}O_{2}$	34.2 ± 5.2 ^c^^d^^e^	33.7 ± 17.1^d^^e^	31.9 ± 4.8 ^a^^e^	31.0 ± 4.2 ^a^^b^	29.6 ± 4.0 ^a^^b^^c^
**Submaximal responses**					
AT (mL^.^ min.^-1^)	1,717 ± 725 ^e^	1,731 ± 690 ^e^	1,611 ± 623 ^e^	1,523 ± 396^e^	1,698 ± 436 ^a^^b^^c^^d^
AT ($\%\dot{V}O_{2}$ peak)	67.4 ± 10.9 ^e^	67.0 ± 13.3 ^e^	67.7 ± 10.0 ^e^	68.7 ± 10.0 ^e^	72.0 ± 8.3 ^a^^b^^c^^d^
AT (% $\dot{V}O_{2}$ peak pred)	71.1 ± 20.4 ^c^	68.9 ± 20.1	66.2 ± 15.8	61.5 ± 12.3 ^a^	63.9 ± 11.4
Lowest $\dot{V}E/\dot{V}CO_{2}$	26.5 ± 3.5	26.5 ± 3.1	26.6 ± 3.1	26.5 ± 2.8	26.7 ± 3.1
**Dynamic physiological responses**					
$\Delta \dot{V}E/\Delta \dot{V}CO_{2}$ (L^.^ min^-1.^ L^.^ min^-1^)	25.7 ± 4.1	25.4 ± 3.8	25.4 ± 3.3	25.3 ± 3.5	25.4 ± 4.4
$\Delta \mathrm{HR}/\Delta \dot{V}O_{2}$ (bpm^.^ L^.^ min.^-1^)	48.2 ± 17.4 ^b^^c^^d^^e^	43.7 ± 15.4 ^a^^c^^d^^e^	42.7 ± 14.0 ^a^^b^^d^^e^	41.7 ± 12.8 ^a^^b^^c^^e^	37.4 ± 10.9 ^a^^b^^c^^d^
$\mathrm{\Delta Vt}/\Delta \ln\dot{V}E$	0.74 ± 0.27 ^e^	0.80 ± 0.28	0.74 ± 0.27	0.71 ± 0.27	0.75 ± 0.28 ^a^
OUES	2,816 ± 900 ^b^^c^^e^	3,121 ± 1.018 ^a^^e^	2,752 ± 844 ^a^^e^	2,762 ± 774 ^e^	2,939 ± 832 ^a^^b^^c^^d^
y-intercept $\dot{V}E/\dot{V}CO_{2}$ (L^.^ min^-1^)	3.72 ± 1.94	3.96 ± 1.95	3.32 ± 2.00	3.72 ± 2.52	3.19 ± 2.54
y-intercept $\Delta \mathrm{HR}/\dot{V}O_{2}$ (bpm)	58.11 ± 14.96	58.55 ± 14.87	58.36 ± 14.65	61.50 ± 13.99	64.76 ± 15.64
y-intercept ΔVt/lnVE (L)	-1.29 ± 0.91	-1.39 ± 0.96	-1.18 ± 1.01	-1.06 ± 1.64	-1.27 ± -1.11

^a^ p ≤ 0.05 vs. Normal weight

^b^ p ≤ 0.05 vs. Overweight

^c^ p ≤ 0.05 vs. Obesity Class I

^d^ p ≤ 0.05 vs. Obesity Class II; and

^e^ p ≤ 0.05 vs. Obesity Class III.

Models adjusted for age, sex, arterial hypertension, diabetes, dyslipidemia, and physical inactivity through self-report. $\dot{V}O_{2}$: pulmonary oxygen uptake; $\dot{V}CO_{2}$: carbon dioxide production; MET: metabolic equivalent of task; AT: anaerobic threshold; R: gas exchange rate; $\dot{V}E$: minute ventilation; MVV: maximum voluntary ventilation; VR: ventilatory reserve; Vt: tidal volume; Rf: respiratory frequency; ipm: inspiration per minute; IC: inspiratory capacity; FVC: forced vital capacity; HR: heart rate; bpm: beat per minute; PuO_2_: oxygen pulse; SBP: systolic blood pressure; DBP: diastolic blood pressure; PETO2: final expiratory oxygen pressure; PETCO_2_: final expiratory pressure of carbon dioxide; $\ln\dot{V}E$: linearized ventilation; OUES: oxygen uptake efficiency slope.

We observed differences mainly between non-obese and obese for gas exchange variables (PETO_2_, PETCO_2_, $\dot{V}E/\dot{V}CO_{2}$, $\dot{V}E/\dot{V}O_{2}$). Among the obese groups, PETO_2_ and $\dot{V}E/\dot{V}O_{2}$ were different only between the classes I and III (**Table 3**).

$\mathrm{\Delta Vt}/\mathrm{\Delta ln}\dot{V}E$ and HR recovery were only altered when comparing normal weight and obese class III. Although PuO2 and OUES differed among the extreme groups (normal weight and obesity class III), we also found differences among the other groups in comparison to them. We found differences in AT when comparing obesity class III with the other groups, for both non-obese and obese. In contrast, all groups presented differences in SBP when compared to normal weight group (**Table 3**).

Regarding the ventilatory (RF, $\dot{V}E$ max, $\dot{V}E/\mathrm{MVV}$, $\dot{V}E/\mathrm{MVV}$) and some cardiovascular (DBP max and DP) variables, we observed inconsistent results (**Table 3**).

**Fig 3:**
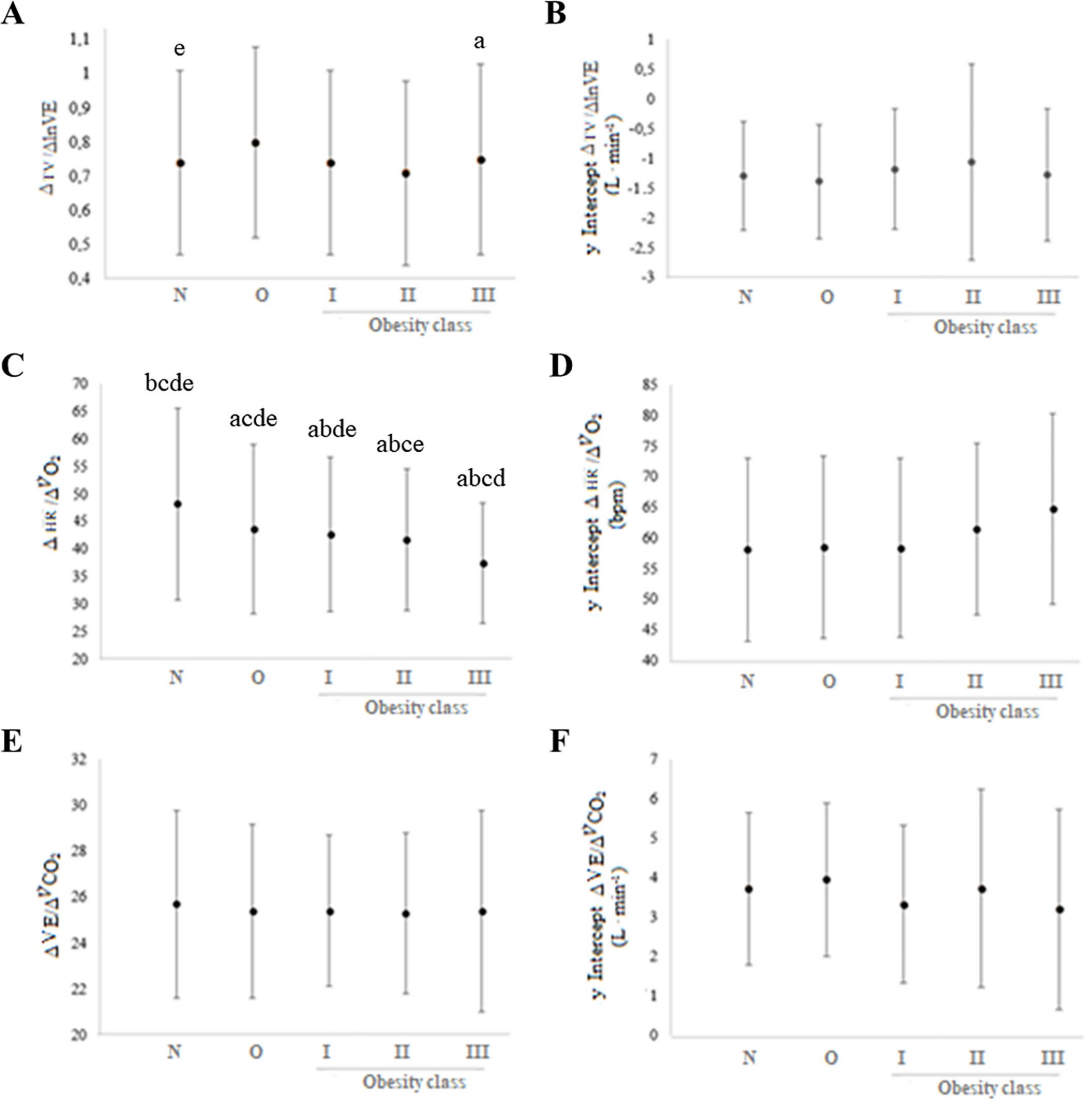
Differences in obesity-related physiological responses in the cardiopulmonary exercise test obtained by using multivariate analysis of variance (n = 1,594). ^a^ p ≤ 0.05 vs. Normal weight; ^b^: p ≤ 0.05 vs. Overweight; ^c^: p ≤ 0.05 vs. Obesity Class I; ^d^ p ≤ 0.05 vs. Obesity Class II; and ^e^ p ≤ 0.05 vs. Obesity Class III. N: normal weight; O: overweight. (A) graphical representation of respiratory pattern; (B) graphical representation of y intercept of respiratory pattern; (C) graphical representation of cardiovascular efficiency; (D) graphical representation of y intercept of cardiovascular efficiency; (E) graphical representation of respiratory efficiency; (F) graphical representation of y intercept of respiratory efficiency. Models adjusted for age, sex, arterial hypertension, diabetes, dyslipidemia, and physical inactivity through self-report. $\dot{V}O_{2}$: pulmonary oxygen uptake; $\dot{V}CO_{2}$: carbon dioxide production; $\dot{V}E$: minute ventilation; Vt: tidal volume; Rf: respiratory frequency; HR: heart rate; bpm: beat per minute; $\ln\dot{V}E$: linearized ventilation; OUES: oxygen uptake efficiency slope.

Lastly, Vt _max_, Vt/VE, Vt/IC, Vt/FVC, Lowest $\dot{V}E/\dot{V}CO_{2}$, $\Delta \dot{V}E/\Delta \dot{V}CO_{2}$, and the y-intercepts of $\dot{V}E/\dot{V}CO_{2}$, did not significantly differ among obese and non-obese participants, as can be seen in Table 3 and Fig 3.

## Discussion

The purpose of present study was to evaluate the influence of adulthood obesity on maximal and submaximal physiological responses during CPET, emphasizing the analysis of submaximal dynamic variables. We observed that both peak and submaximal responses are influenced by obesity, to a greater or lesser degree. Maximal cardiovascular and metabolic variables were the most affected responses regarding obesity, but, for some ventilatory and cardiovascular responses, our findings were inconclusive due to inconsistent results. For other variables, mainly gas exchange responses, we found differences when comparing non-obese and obese or related to severity of obesity. However, our main finding was the non-significant influence of obesity on ventilatory efficiency variables (Lowest $\dot{V}E/\dot{V}CO_{2}$, $\Delta \dot{V}E/\Delta \dot{V}CO_{2}$ and the y-intercepts of $\dot{V}E/\dot{V}CO_{2}$), which may suggest best prognostic value regardless of overweight and obesity. To our knowledge, this is the first study to investigate the influence of obesity, according to severity and adjusted by the main cardiovascular risk factors, on the dynamic physiological responses obtained in the CPET in a robust sample.

As expected, maximal cardiovascular responses (HR_max_, HR% _pred_, HR _reserve_) and cardiovascular efficiency ($\mathrm{\Delta HR}/\Delta \dot{V}O_{2}$) were altered along with overweight and obesity. Our findings are corroborated by previously studies that showed rapidly increased cardiac output and unfavorable biomechanics leading to cardiovascular inefficiency.

In contrast, HR recovery and PuO_2_ seems to be related to the severity of obesity. Similarly, previous studies have observed a slow HR_recovery_ in obese individuals [20, 21] and its improvement after weight loss [22]. In addition to relevant methodological differences, Carnethon et al. [20] characterized the sample according to normal weight, overweight and obese participants and presented proportionally less obese subjects when compared to our study. Thus, our study adds to literature by showing that this difference occurs in morbidly obese participants. Our findings also suggest that HR_recovery_ ≥ 22 bpm, as a parasympathetic normal response, can be applied to interpret results from normal weight, overweight and obesity classes I and II. Therefore, we recommend the use of HR_recovery_ in CPET analysis and interpretation since it is a simple, reproducible, and easy-to-access variable, besides having a high mortality predictive value, association with increased cardiovascular risk [23, 24] and additional value to the CRF prognosis [25].

Regarding PuO_2_, literature remains scarce related to obesity and there is a lack of value to interpret this response. In the present study, morbidly obese had higher peak values of PuO_2_, which may be possible explained by the high consumption of $\dot{V}O_{2}$ per workload unit presented by obese individuals. Similar results were observed by Serés et al. [26], who attributed these finds to a greater stroke volume related to an increased weight bearing in obese individuals. Previous study suggests that PuO_2_ kinetics must be altered in cases of less cardiovascular efficiency, but its analysis requires visual inspection. Further studies interested in analyzing PuO_2_ must include the additional analysis of its kinetics.

As expected, obesity influenced maximal $\dot{V}O_{2}$ in agreement with previous studies. The average energy expenditure during the CPET, represented by the MET, is higher in obese individuals due to a high energy expenditure to perform activities related to the excess of weight, which, according to Delany et al. [27], could mask the lower levels of physical activity performed by these subjects.

Regarding submaximal responses, AT was only significantly different in morbid obesity. Similarly, previous study found that AT was not different among obese and normal weight women [28]. However, we observed that AT in morbidly obese represents a high % $\dot{V}O_{2\mathrm{peak}}$, instead of % $\dot{V}O_{2\mathrm{peak}}$ predicted, which may be explained by two main factors. First, the obese subjects are less physically active and conditioned, leading to the proximity of AT and peak $\dot{V}O_{2}$, due to an early CPET interruption. Secondarily, we used Hansen’s equation to estimate the $\dot{V}O_{2\max}$ [29], which can overestimate this value and, hence, underestimate the subjects by providing an inadequate ramp that also contributes to a possible early CPET interruption.

We showed that OUES significantly differed in morbidly obese, but it should be carefully analyzed. Previous findings observed that obese individuals consume more O_2_ at the same effort intensity [30]. Although a promising dynamic physiological response [31, 32], OUES is dependent to exercise intensity since it can underestimate $\dot{V}O_{2\max}$ at low intensity, but overestimate at high intensity effort [30]. Additionally, OUES present an important interindividual variability [33] that can compromise its use in clinical practice. Thus, OUES interpretation may be difficult [13] despite its potential role as prognostic value for mortality regardless BMI.

In our study, we observed that both the Vt/IC ratio, which can be used to help determine the ventilatory reserve, and the Vt/FVC ratio did not present significant differences between non-obese and obese participants. Our results agree with previous studies on obese individuals with COPD, which observed that obesity did not worsen dyspnea or exercise performance in these individuals [34, 35]. A possible explanation found are related to alterations in the elastic properties of the lungs, increased intra-abdominal pressure, decreased pulmonary hyperinflation, and preserved inspiratory capacity during effort [34, 35].

Related to $\Delta \dot{V}E/\Delta \dot{V}CO_{2}$, Neder et al. [36] observed a greater ventilatory efficiency in individuals in the early stages of COPD and flattening of this relationship with the progression of disease severity. Supposedly, these finds indicate better ventilatory efficiency in these patients, but physiologically, this efficiency should be worsened in these individuals due to the impairment of limited ventilatory mechanics combined with increased CO_2_ pressure (PETCO_2_) [36]. Similarly, obese individuals also have limited ventilatory mechanics due to central obesity, which may reduce the compliance of the lungs and, therefore, may lead to a restrictive ventilatory pattern during effort [37]. However, we did not observe an altered response in $\Delta \dot{V}E/\Delta \dot{V}CO_{2}$, which was an unexpected finding possibly associated with the use of BMI classification since each class can range from 2 to 3 times in visceral adiposity [38].

Concomitant with this paradoxical result, there was an increase in the value of the y-intercept of $\Delta \dot{V}E/\Delta \dot{V}CO_{2}$ related to the severity of obesity. It is possible that the y-intercept of the $\Delta \dot{V}E/\Delta \dot{V}CO_{2}$ is more sensitive than the relationship slope to indicate the progression of ventilatory inefficiency in patients with COPD during effort [36]. Both the y-intercept and the $\Delta \dot{V}E/\Delta \dot{V}CO_{2}$ slope remained unchanged in our study. Therefore, these indexes have a promising role in prognostic and diagnostic related to ventilatory inefficiency.

In addition, the lowest $\dot{V}E/\dot{V}CO_{2}$ was also not different across non-obese and obese groups. Considering the great clinical interest for early identification of respiratory disorders and incipient respiratory diseases, any change found in $\dot{V}E/\dot{V}CO_{2}$ suggests a ventilatory inefficiency during effort and should be further investigated.

The fat deposits in the mediastinum and the abdominal cavities due to obesity, as previously discussed, reduce the compliance of the lungs and, therefore, may lead to a restrictive ventilatory pattern during effort [37]. However, ventilatory pattern (ΔVt/ $\mathrm{\Delta ln}\dot{V}E$) showed no significant influence of obesity. In this context, lower values of this ratio, indicative of tachypnea respiratory pattern, would most likely suggest an incipient ventilatory disorder regardless of obesity severity. Recently, Chao et al. [39] compared spirometry and CPET to detect incipient ventilatory disorders in workers exposed or not to occupational particulate matter. In addition to best sensibility of CPET, the $\mathrm{\Delta Vt}/\mathrm{\Delta ln}\dot{V}E$ was one of the most useful variables being able to differentiate exposed from unexposed workers. Therefore, our results reinforce the clinical usefulness and prognostic value of this submaximal ratio.

As expected, the increment (treadmill speed and grade) decreased with obesity severity. The treadmill is an extremely popular ergometer in cardiology and sports medicine. A disadvantage of this ergometer is the workload, which cannot be accurately measured, since it depends on other factors (sustained body mass, anthropometry, and biomechanical aspects of gait) [40, 41]. In comparison to cycle ergometer, using a treadmill leads to greater cardiorespiratory stress which occurs due to the greater muscle mass involved in the exercise, venous return, HR, cardiac output and $\dot{V}O_{2}$ for the same workload [40, 41]. In addition, these physiological responses enable a best diagnosis of electrocardiographic changes, especially at maximal effort. Finally, using a treadmill provides greater representativeness of daily living activities (walking and running) [40–42].

The present study has some limitations that must be addressed. It is important to consider that the use of the treadmill implies a lack of objective workload. However, we registered the speed and grade during effort, which minimized this limitation. Our main limitation was the estimation of the peak of $\dot{V}O_{2}$ by using Hansen’s equation that can overestimate this value in obese subjects [29]. In order to deal with this possible limitation, we excluded submaximal effort tests in addition to rigorous analysis of R, HR, perceived subjective exertion and exercise duration. Despite the fact that a convenience sample was an important limitation, our large sample was appropriate, including a sufficient sample in each group of non-obese and obese participants. Additionally, we adjusted our analysis for the main confounders. Finally, although our findings are clinically relevant, especially to improve CPET interpretation, we point out that these aforementioned parameters present a wide variability. Thus, despite our results, their implementation in clinical practice routine can still be challenging and needs to be addressed in future research.

Our results have practical implications for public health. Obese individuals, most often, have increased cardiovascular risk, and CPET is the gold standard for assessing the CRF related to numerous comorbidities. However, the maximal variables must be influenced by motivation besides being related to several biomechanical aspects, especially relevant regarding obesity. In contrast, submaximal data are more responsive to treatment and exercise training in comparison to maximal variables and, hence, may be useful in early diagnosis of limitations in exercise tolerance and development of strategies to health promotion and obesity-related prevention. Therefore, the measurement and interpretation of submaximal and dynamic responses in CPET must be encouraged in clinical practice routine for both non-obese and obese patients. Based on our findings, future investigations should analyze the variables presented here to reinforce their clinical value in obese individuals in different settings and age range based on our findings. It is also of our interesting that prospective studies assess and confirm the prognostic value of the studied variables, mainly $\dot{V}E/\dot{V}CO_{2}$, $\Delta \dot{V}E/\Delta \dot{V}CO_{2}$ and the y-intercepts of $\Delta \dot{V}E/\Delta \dot{V}CO_{2}$, in obese individuals, in addition to its use in clinical practice routine.

## Conclusion

Obesity expressively influences maximal and submaximal CPET physiological responses, mainly cardiovascular and metabolic. Only a few variables presented inconsistent findings, which can reduce its applicability in the test interpretation regarding overweight and obese subjects. In contrast, the prognostic values of the main ventilatory efficiency variables, e.g., Vt max, $\mathrm{Vt}/\dot{V}E$, Vt/IC, Vt/FVC, lowest $\dot{V}E/\dot{V}CO_{2}$, $\Delta \dot{V}E/\Delta \dot{V}CO_{2}$, and the y-intercepts of $\dot{V}E/\dot{V}CO_{2}$ remained unchanged and, therefore, has the best prognostic value regardless of obesity. These dynamic responses are independent of maximum effort and may be useful in detecting incipient ventilatory disorder. Our results present great practical applicability in identifying exercise limitation, regardless of BMI.

## List of abbreviations

AT: Anaerobic threshold
BMI: Body Mass Index
Bpm: Beat per minute
COPD: Chronic obstructive pulmonary test
CPET: Cardiopulmonary exercise test
CRF: Cardiorespiratory fitness
DBP: Diastolic blood pressure
DP: Double product
FVC: Forced vital capacity
HR: Heart rate
HR_max_: Maximum heart rate
HR_reserve_: Reserve heart rate
HR%_pred_: Percent of predicted heart rate
IC: Inspiratory capacity
ipm: Inspiration per minute
kg: Kilogram
km: Kilometer
L: Liter
$\ln\dot{V}E$: Linearized minute ventilation
$\log\dot{V}E$: Logarithm of minute ventilation
max: Maximum
MET: Metabolic Equivalent of Task
min: minutes
mL: milliliter
mmHg: millimeter of mercury
ms: millisecond
MVV: Maximum voluntary ventilation
OUES: Oxygen uptake efficiency slope
PaCO_2_: Carbon dioxide partial blood pressure
PaO_2_: Partial oxygen blood pressure
PETCO_2_: Final expiratory pressure of carbon dioxide
PETO_2_: Final expiratory pressure of oxygen
PuO_2_: Oxygen pulse
R: Gas exchange rate
Rf: Respiratory frequency
SBP: Systolic blood pressure
TLC: Total lung capacity
Vt: Tidal volume
$\dot{V}{\mathrm{CO}}_{2}$: Carbon dioxide production
$\dot{V}E$: Minute ventilation
$\dot{V}O_{2}$: Pulmonary oxygen uptake
$\dot{V}O_{2\max}$: Maximum pulmonary oxygen uptake
$\dot{V}O_{2\max\%\mathrm{pred}}$: Predicted percentage of maximum pulmonary oxygen uptake
VR: Ventilatory reserve
